# Supplementary material for: Inferring selection effects in SARS-CoV-2 with Bayesian Viral Allele Selection
Source: PLoS Genet. 2022 Dec 12;18(12):e1010540. doi: 10.1371/journal.pgen.1010540 (PMC9779722; doi:10.1371/journal.pgen.1010540)
Supplement: Attachment — Submitted filename: bvas reviewer response.pdf [file pgen.1010540.s004.pdf]

We thank both reviewers for taking the time to carefully read our manuscript and for their constructive comments. We are gratified that both reviewers found the manuscript well-written and ready for publication after minor revisions. We respond to reviewer comments in detail below and mark changes made to the manuscript in red (both here and in the marked revised pdfs). A summary of editorial/latex changes made to adhere to PLOS guidelines is included at the end.

Reviewer #1:

Line 28: Add computationally before expensive (assuming this is what you mean) and remove from line 30.

Done

Line 53: Give a brief I sentence definition of neutral/non-neutral alleles as this is an important aspect of the paper.

We have rewritten this sentence as follows:

However, unlike PyR_0 MAP does not assume that most alleles are approximately neutral, i.e. that most alleles have little or no effect on viral fitness. As a result MAP risks inferring non-negligible selection effects for implausibly many alleles.

Lines not given (2nd sentence of Sec 2.1): In methods the sentence begging 'Since SARS-CoV2 and SARS-CoV-2 are know…' Split into two sentences as is somewhat hard to read in its current format.

We have rewritten this sentence as follows:

SARS-CoV and SARS-CoV-2 are known to exhibit super-spreading (Lloyd-Smith et al., 2005; Althouse et al., 2020) whereby a minority of infected individuals cause the majority of secondary infections. To account for this behavior the number of secondary infections is assumed to be governed by a Negative Binomial distribution, which has a large variance for small values of the dispersion parameter k.

Lines 390-391: Spurious alleles you mean not SARS-CoV-2, just be more specific.

We have rewritten as "200 synthetic (and therefore spurious) alleles"

Line 444/Table1: BA4 does not actually appear in the table so remove from the sentence or add to the table.

We have removed BA.4 from the discussion in lines 444ff (and fixed a small typo).

Table3/Lines469: would be useful to have a column specifying, for those mutations in spike, where in the spike protein they are located. Either as simple as RBD/non-RBD or more specific as you pick up on the FC site later.

Thanks for the suggestion. We have added a RBD annotation column to both subtables in Table 3 that looks as follows.

	Mutation	RBD	PIP	Beta
**1**	S:R346K	Yes	1.0000	0.371 ± 0.048
**2**	S:L452R	Yes	1.0000	0.435 ± 0.103
**3**	S:E484K	Yes	1.0000	0.386 ± 0.097
**4**	S:N501Y	Yes	1.0000	0.364 ± 0.118
**5**	M:I82T		1.0000	0.335 ± 0.068
**6**	S:D614G		1.0000	0.339 ± 0.119
**7**	S:A222V		1.0000	0.170 ± 0.058
**8**	S:P681H		1.0000	0.216 ± 0.103
**9**	S:S477N	Yes	0.9999	0.231 ± 0.146
**10**	S:P681R		0.9999	0.338 ± 0.146
**11**	ORF1b:K1383R		0.9994	-0.106 ± 0.045
**12**	S:N440K	Yes	0.9992	0.296 ± 0.126
**13**	ORF1b:H1087Y		0.9978	0.165 ± 0.057
**14**	ORF1a:A2529V		0.9977	0.097 ± 0.039
**15**	S:H655Y		0.9939	0.328 ± 0.154
**16**	ORF1a:A2554V		0.9544	-0.115 ± 0.060
**17**	S:N679K		0.9500	0.321 ± 0.233
**18**	ORF1a:T3255I		0.9468	0.196 ± 0.145
**19**	S:T859N		0.9454	0.236 ± 0.182
**20**	N:D3L		0.9145	0.238 ± 0.166
**21**	S:L452Q	Yes	0.8827	0.259 ± 0.243
**22**	S:N764K		0.8334	0.267 ± 0.302
**23**	M:A63T		0.8127	0.236 ± 0.263
**24**	S:S371F	Yes	0.8065	0.245 ± 0.289
**25**	S:N969K		0.7888	0.250 ± 0.315

Line 500: does 'S-gene' include RBD or is it exclusive of RBD given that RBD has it's own category.

It is inclusive. For clarity we have amended the first sentence of the caption of Figure 10 as follows:

We aggregate BVAS β estimates into S-gene (including the RBD), RBD (S gene receptor-binding domain), and non-S-gene components…

4.4 Backtesting: Would be interesting to include omicron BA.4 and BA.5 analysis here, if it is possible to do in a timely manner. This would also make the paper more relevant to the current COVID-19 landscape.

Backtesting is quite computationally expensive and we are unsure what we might hope to learn from additional backtesting analyses. We do agree, however, that it would be valuable to include BVAS inferences based on more recent data in the revised manuscript.

Consequently we have included BVAS inference results obtained from 8.5 million sequences from 151 regions collected through August 10th, 2022 (i.e. including ~4 months/2M samples of additional data compared to our main analysis). In particular we have added the following table (which is a direct analog of Table 1 in the main text) to the supplement as Table S3:

	Lineage	Growth Rate		Lineage	Growth Rate
**1**	BF.7	9.475 ± 0.693	**11**	BF.9	8.916 ± 0.678
**2**	BF.11	9.457 ± 0.687	**12**	BA.5.2.4	8.915 ± 0.677
**3**	BA.5.2.3	9.088 ± 0.679	**13**	BF.6	8.915 ± 0.677
**4**	BA.5.2.2	9.040 ± 0.695	**14**	BF.4	8.915 ± 0.677
**5**	BA.4.6	8.926 ± 0.686	**15**	BA.5.2.5	8.914 ± 0.678
**6**	BA.4.1.8	8.923 ± 0.686	**16**	BF.2	8.913 ± 0.678
**7**	BF.5	8.919 ± 0.678	**17**	BF.8	8.911 ± 0.679
**8**	BF.3.1	8.918 ± 0.679	**18**	BA.5.2	8.874 ± 0.788
**9**	BF.3	8.917 ± 0.678	**19**	BE.1.1	8.811 ± 0.687
**10**	BF.10	8.917 ± 0.678	**20**	BE.1	8.809 ± 0.687

In addition we have added the following two subtables (which are a direct analog of the two subtables in Table 3 in the main text) as Table S4:

	Mutation	RBD	PIP	Beta		Mutation	RBD	PIP	Beta
**1**	S:L452R	Yes	1.0000	0.609 ± 0.140	**1**	S:L452R	Yes	1.0000	0.609 ± 0.140
**2**	S:R346T	Yes	1.0000	0.542 ± 0.119	**2**	S:L452Q	Yes	1.0000	0.490 ± 0.087
**3**	S:F486V	Yes	1.0000	0.525 ± 0.121	**3**	S:R346K	Yes	1.0000	0.348 ± 0.045
**4**	S:L452Q	Yes	1.0000	0.490 ± 0.087	**4**	S:E484K	Yes	1.0000	0.360 ± 0.087
**5**	S:L452M	Yes	1.0000	0.454 ± 0.105	**5**	S:R346T	Yes	1.0000	0.542 ± 0.119
**6**	S:N969K		0.9946	0.411 ± 0.205	**6**	S:L452M	Yes	1.0000	0.454 ± 0.105
**7**	S:Q954H		0.9944	0.410 ± 0.205	**7**	S:N501Y	Yes	1.0000	0.363 ± 0.113
**8**	S:N764K		0.9952	0.404 ± 0.198	**8**	S:S477N	Yes	1.0000	0.309 ± 0.114
**9**	S:N679K		0.9986	0.395 ± 0.176	**9**	S:F486V	Yes	1.0000	0.525 ± 0.121
**10**	S:V213G		0.9939	0.391 ± 0.205	**10**	ORF9b:D16G		1.0000	0.212 ± 0.070
**11**	ORF1b:I1566V		0.9811	0.375 ± 0.219	**11**	M:I82T		1.0000	0.346 ± 0.073
**12**	E:T9I		0.9807	0.375 ± 0.119	**12**	ORF1a:T3255I		1.0000	0.300 ± 0.090
**13**	S:N501Y	Yes	1.0000	0.363 ± 0.113	**13**	S:P681H		1.0000	0.209 ± 0.093
**14**	S:E484K	Yes	1.0000	0.360 ± 0.087	**14**	S:I68T		1.0000	0.305 ± 0.104
**15**	S:R346K	Yes	1.0000	0.348 ± 0.045	**15**	S:T76I		1.0000	-0.231 ± 0.083
**16**	M:I82T		1.0000	0.346 ± 0.073	**16**	M:D3N		1.0000	0.322 ± 0.070
**17**	S:R408S	Yes	0.9812	0.342 ± 0.200	**17**	S:N440K	Yes	1.0000	0.314 ± 0.121
**18**	M:D3N		1.0000	0.322 ± 0.070	**18**	S:K417N	Yes	0.9999	-0.261 ± 0.106
**19**	S:P681R		0.9967	0.318 ± 0.170	**19**	ORF1b:S959P		0.9999	-0.204 ± 0.075
**20**	S:N440K	Yes	1.0000	0.314 ± 0.121	**20**	ORF1a:A2529V		0.9995	0.094 ± 0.035
**21**	S:S477N	Yes	1.0000	0.309 ± 0.114	**21**	S:A222V		0.9995	0.193 ± 0.070
**22**	S:I68T		1.0000	0.305 ± 0.104	**22**	ORF1a:A2554V		0.9994	-0.120 ± 0.033
**23**	ORF1a:T3255I		1.0000	0.300 ± 0.090	**23**	ORF1b:K1383R		0.9990	-0.094 ± 0.042
**24**	ORF1b:G662S		0.9396	0.289 ± 0.213	**24**	S:N679K		0.9986	0.395 ± 0.176
**25**	ORF9b:P10S		0.9949	0.288 ± 0.122	**25**	S:K417T	Yes	0.9974	0.224 ± 0.127

We have also uploaded raw inference results to our GitHub repository here: https://github.com/broadinstitute/bvas/tree/main/paper/08.10.22

We anticipate uploading updated inference results on a ~monthly basis going forward. For example: https://github.com/broadinstitute/bvas/tree/main/paper/09.22.22

In particular we include lineage-level results (growth_rates_summary.csv) and allele-level results (allele_summary.csv). We have also pointed to these new results in the main text by adding footnote 9 at the beginning of Sec 4.1. The lineage- and mutation-level inferences are remarkably stable between runs of the model and highlight the robustness of BVAS.

In obtaining these updated inference results we have lightly adapted our strategy for estimating effective population sizes. We believe this strategy is better suited for automation (we are currently working towards running BVAS at a more frequent cadence). We describe this new strategy at the end of Sec. S14.1.5:

We also describe an alternative strategy for computing the effective population size that we use in our analysis of SARS-CoV-2 surveillance data obtained through August 10th 2022. As above we compute effective population size estimates for each region using Eqn. 36. We then partition the regions into two buckets based on the median effective population size: i) those whose ν exceeds the median; and ii) those whose ν is less than or equal to the median. That is we partition regions into lower and upper 50% quantiles based on their estimated effective population sizes. We then compute y and Λ using the median ν for the upper bucket and the minimum ν (i.e. the minimum among all regions) for the lower bucket. We call this effective population size estimation strategy the 'two-bucket' strategy. It has the following nice properties: i) it is parameter free; ii) it weighs data from well-sampled regions like the UK more than poorly-sampled regions (though only by a moderate O(1) factor); and iii) it is still conservative in that we generally expect to underestimate the true effective population size in each region.

Reviewer #2:

1. Sequencing rates and surveillance efforts are highly non-uniform across different nations. Perhaps the author should discuss how this may affect BVAS and how realistic is its assumption of an i.i.d. sampling rate.

As discussed in Sec. 2.7 and Sec. S8 the effect of i.i.d. sampling (at least within a well-mixing region) is to renormalize the effective population size. Furthermore as discussed in S14.1.4 our data pre-processing filters drop regions and time windows where the number of surveillance samples is low. Moreover in S14.1.5 we discuss how we infer a single global effective population size. With this background in mind, we have added the following text to Sec. S12, where we discuss some of the limitations of BVAS:

Additional bias and/or reduced statistical power may result from our use of a global effective population size, which implicitly assumes that the sampling rate does not vary wildly from region to region. In particular by (very likely) *underestimating* the effective population size in well-sampled regions like the UK we lose statistical power, although we note that by using a smaller effective population size we expect our inferences to be less sensitive to noise in the data and thus more robust. Moreover by using a single effective population size we implicitly assume that the sampling rate is approximately uniform within each region. We do not expect a non-uniform sampling rate within a given region to bias results provided that the region is well mixed. Biased inferences may result, however, if a region is poorly mixed and exhibits non-uniform sampling, as this combination of violations of our modeling assumptions may overemphasize the importance of local fluctuations in allele frequencies.

2. Simulated datasets use a sampling rate between 1-64%. It is likely that the COVID-19 sampling rate is a lot lower than 1%. Why not evaluate the results for smaller sampling rate values as well?

As discussed in Sec. 2.7 and Sec. S8 the effect of lowering the sampling rate is to renormalize the effective population size, in particular to reduce it. As such Figure S4 can more generally be interpreted as an investigation of the robustness of our results to reduced effective population sizes. This reduction could result from a reduced sampling rate but could also result from e.g. a reduced dispersion parameter k (i.e. more super-spreading).

As discussed in Sec. 3.1 and Sec. S13 our simulation parameters were chosen to reproduce effective population sizes comparable to those we see in the real data. Our default simulation settings consider an underlying population of 10^4 infected individuals and a sampling rate of 1%, however we could have just as well chosen 10^5 infected individuals and a sampling rate of 0.1% and observed nearly identical results. As such we believe that varying the sampling rate by a factor of 64 in Figure S4 is already enough to demonstrate the robustness of our results to sampling (or indeed changes in effective population size due to other factors).

3. Simulated dataset use a small reproduction number range of 0.9 to 1.1. The range of R0 for SARS-CoV-2 is much higher. How does BVAS perform when this range is increased?

There are several interlocking reasons why we made this choice in our simulations. The first is that we wanted to assess our sensitivity to small effect sizes. As you would expect whenever the “hit rate” is below 1.0 in Figure 1 it is typically because the alleles with the smallest effect sizes (e.g. 0.01 or 0.02) failed to be identified. The second reason is that our simulations use the discrete time branching process described in Sec. 2.1. If we consider a significantly larger range of reproduction numbers, we invariably see explosive dynamics where the number of infected individuals increases exponentially throughout the 26 time steps of our simulation. This results in effective population sizes that vary wildly across time, which is not what we see in the real data. By choosing a more moderate range of reproduction numbers we observe effective population sizes that range from ~25 to about ~140 from region to region, which is in line with what we observe in the real data.

In order to accommodate larger ranges of reproduction numbers our simulation would need to explicitly model the fact that human behavior tends to change when confronted with explosive viral transmission (e.g. self-isolation or lockdown measures), which then dampens the explosive growth (at least for a time). While such an analysis might be informative, we decided not to pursue this approach because we did not want to include additional (probably ad hoc) assumptions in our simulation. Overall, our simulations confirm our expectation that alleles with large effect sizes will be easier to identify than alleles with small effect sizes, simply because large effects lead to a deterministic drift component that stands out more clearly on top of the background of stochastic fluctuations in allele frequency.

4. What is the value of sigma-Laplace for data in Fig. 1.?

This is discussed in Sec. S13.3 (line 296), which we reproduce here:

> For Laplace we use a regularization scale of $\sigma^{Laplace} = 0.01$.

This happens to be the best choice for A = 3000 alleles and 30 regions (see Figure 3) but is probably suboptimal for our simulation with 100 regions (of course the whole point of these analyses is that it is difficult to know how to set $\sigma^{Laplace}$ a priori).

5. For Fig. 4, why is the hit rate not optimal at #non-neutral sites=10?

This is briefly discussed in lines 317ff in Sec. 3.3:

> The effect of choosing small S is to be more conservative; in particular some weak effects at the threshold of discovery may be assigned small PIPs.

One of the features of the sparsity assumption that underlies BVAS is that BVAS tends to be conservative when it comes to effects at the threshold of discovery. The smallest effect sizes in our simulations are 0.01 and -0.01. Thus even if S (the expected number of non-neutral alleles a priori) is set to the true number of causal alleles, those alleles with small effect sizes may be assigned small PIPs. This is somewhat unfortunate but is more or less inevitable, since some sort of regularization is required to avoid assigning large PIPs to lots of spurious alleles. In Figure 4 we get better hit rates with a higher S (S >> 10) but this is because in this case the model is more or less perfectly well specified (since our diffusion-based likelihood is derived from the discrete branching process that underlies our simulations) and so it's safe to be less conservative. For real world data where model mis-specification is more of a concern, we would expect that choosing a very large S would lead to lots of spurious hits.

Summary of editorial/latex changes:

- The marked-up copy of our revised manuscript uses the Latex style used for the original submission for ease of comparison
- The clean copy of our revised manuscript uses the PLOS style
- We have added an Author summary
- We have retained (sub)section numbers as suggested by latex@plos.org
    - *"PLOS generally prefers un-numbered sections, but we will make exceptions if removing the numbering will cause confusion to the reader."*
- We have used PACE to convert all figures in the main text from pdfs to tiffs (Fig1.tiff, etc). We include the pdfs in our submission so the latex can be compiled correctly, although these won't be needed if PLOS uses tiff everywhere.
- We have renumbered supplementary figures and tables as A, B, …, Z, AA, BB, … as suggested by latex@plos.org
- Tables in the main text in the original submission were pdfs. These have been converted to latex tables.
- We have added the following sections to the end of the main text: Author Contributions, Supporting Information
- We have added macros \Sfigref, \Stabref, etc. to refer to figures and tables in the Supplementary information. Thus it should be easy for PLOS to modify how these references appear.
- We have added bold titles to figures and tables in the main text (e.g. **Table 1. Top 20 fittest SARS-CoV-2 lineages.**)
- Where possible we have updated references from pre-prints to published manuscripts
